# Policy versus practice: a community-based qualitative study of the realities of pharmacy services in Nunavut, Canada

**DOI:** 10.1186/s40545-015-0043-5

**Published:** 2015-09-21

**Authors:** Sandra J. Romain, Jillian C. Kohler, Kue Young

**Affiliations:** Department of Anthropology, University of Toronto Scarborough, 1265 Military Trail, M1C 1A4, Toronto, Ontario Canada; Department of Anthropology, University of Toronto, 19 Russell Street, M5S 2S2, Toronto, Ontario Canada; Associate Professor and Director, Global Affairs, Leslie Dan Faculty of Pharmacy, University of Toronto, 144 College Street, M5S 3M2, Toronto, Ontario Canada; School of Public Health, University of Alberta, 11405-87 Avenue, T6G 1C9, Edmonton, Alberta Canada

**Keywords:** Nunavut, Arctic, Pharmacy, Medication, Prescription, Policy, Remote

## Abstract

**Objectives:**

Nunavut is an Arctic territory in Canada subject to many social, economic and health disparities in comparison to the rest of the nation. The territory is affected by health care provision challenges caused by small, geographically isolated communities where staffing shortages and weather related access barriers are common concerns. In addition to national universal healthcare, the majority of the inhabitants of Nunavut (~85 %) are Inuit beneficiaries of no-charge pharmaceuticals provided through federal and/or territorial budgetary allocations. This research examines how existing pharmaceutical administration and distribution policies and practices in Nunavut impact patient care.

**Methods:**

This grounded theory research includes document analysis and semi-structured interviews conducted in 2013/14 with patients, health care providers, administrators and policy makers in several communities in Nunavut. Thirty five informants in total participated in the study. Interviews were audiotaped, transcribed and analyzed with qualitative data analysis software for internal consistency and emerging themes.

**Results:**

Four distinct themes emerge from the research that have the potential to impact patient care and which may provide direction for future policy development: 1) tensions between national versus territorial financial responsibilities influence health provider decisions that may affect patient care, 2) significant human resources are utilized in Community Health Centres to perform distribution duties associated with retail pharmacy medications, 3) large quantities of unclaimed prescription medications are suggestive of significant financial losses, suboptimal patient care and low adherence rates, and 4) the absence of a clear policy and oversight for some controlled substances, such as narcotics, leaves communities at risk for potential illegal procurement or abuse.

**Conclusions:**

Addressing these issues in future policy development may result in system-wide economic benefits, improved patient care and adherence, and reduced risk to communities. The interview informants who participated in this research are best positioned to identify issues in need of attention and will benefit the most from policy development to address their concerns.

## Introduction

### Profile of Nunavut

Stark contrasts are commonplace in the Arctic, where light and land, and tradition and modernity mix to create unique landscapes. The Arctic territory of Nunavut is the largest, least populated and newest territory in Canada, created from negotiations between the federal government and the Aboriginal Inuit inhabitants of the region in 1999; as such, 85 % of the population of Nunavut are Inuit [1]. In contrast to Canada’s high income and development measures, Nunavut demonstrates substantially deficient indicators for health and social and economic development. Nunavut’s epidemiological profile includes disproportionately high levels of many infectious and chronic diseases, as well as high age-standardized mortality rates (603 in Nunavut versus 259 in Canada, per 100,000), suicide rates (56.9 in Nunavut versus 10.4 in Canada, per 100,000) and infant mortality rates (16.1 in Nunavut versus 5.1 in Canada, per 1,000 live births) [2]. Life expectancy at birth differentials between Nunavut and the rest of Canada are seven years for women (76 years versus 83 years, respectively) and nine years for men (69 years versus 78 years, respectively) [3]. Nunavut is also affected by social issues such as poverty (the Nunavut average household income is 10 % lower than the Canadian average while household food expenditures are three times the national average), unemployment (46.2 % employment rate for working-age Inuit), food insecurity (>70 % of households in Nunavut versus 9 % in Canada), and low educational attainment (27.5 % high school graduation rate from 1999 to 2008) [4]. Additionally, high birth rates (2.97 children per woman versus 1.61 per woman for Canada in 2011) and overcrowded housing (52.7 % live in crowded housing versus 3 % in the rest of Canada) have significant impacts on health measures and service needs [5, 6]. The Community Well-Being Index (CWB) is a composite measure developed by the Canadian Department of Indian and Northern Affairs, which considers income, education, housing and labour-force participation. In the Canadian northern territories, the average CWB score for Aboriginal communities is 62.0, whereas for the rest of Canada the average CWB score is 74.2 [3].

Health care in Nunavut is delivered in Community Health Centres (CHCs) through a nurse-led primary care delivery model with services being provided predominantly by community health nurses (CHNs); the vast majority of CHNs are non-Inuit originally from the south [7]. The territory of Nunavut is divided administratively into three regions: the Kivalliq in the southwest, the Kitikmeot in the northwest, and the Qikiqtaaluk in the east. The two western regions each have a Regional Health Centre with limited in-patient capacity and extended diagnostic testing services (Rankin Inlet in the Kivalliq and Cambridge Bay in the Kitikmeot). The territorial hospital (Qikiqtani General Hospital) is located in the capital of Iqaluit in the Qikiqtaaluk. Each of the remaining 22 communities is serviced by a CHC. Regional Health Centres are distribution hubs for CHC medications, with Qikiqtani General Hospital serving centralized ordering and administration roles. At the CHCs, medications are compounded and/or dispensed by health providers from bulk containers into smaller amounts for individual patients as required. For prescribed medications that are not provided by the CHCs, there are five retail pharmacies within the territory: two in Iqaluit, two in Rankin Inlet and one in Cambridge Bay. In communities without a retail pharmacy, all prescribed medications are dispensed by the closest regional retail pharmacy, flown into the community and delivered directly to the CHC for distribution to community members.

This disparity, of an underdeveloped territory situated within a nation enjoying high development indicators more generally, creates a unique contrasting backdrop for pharmaceutical service provisions. Provinces and territories are responsible for the provision of health services in accordance with Canada’s publicly funded universal health care program. The Government of Nunavut (GN) funds primary and hospital care and inpatient pharmaceuticals in accordance with the GN Drug Formulary Manual. These pharmaceuticals (referred to as wardstock) are defined as those that are used “at the time of care in a health centre, hospital or public health unit, as well as may be provided in a small supply to treat patients for a short period after the patient returns home” [8]. Canada’s universal health care program does not extend to outpatient prescription pharmaceuticals or over the counter (OTC) medications. However, as the majority of the population of Nunavut are Inuit, they are beneficiaries of the Non-Insured Health Benefits (NIHB) Program for First Nations and Inuit. These benefits encompass many health-related goods and services including 100 % coverage for most outpatient prescription pharmaceuticals and many OTCs not covered by the GN; these medications are dispensed from retail pharmacies [9]. This broad sweeping medical coverage is the result of historical treaties signed between Aboriginal groups and the Canadian federal government in the 1870s which included references to a “medicine chest” clause agreeing to provide medical care and medicines to the Aboriginal peoples who had signed the treaties [10].

### Financial expenditures

Federal funding for the NIHB program is allocated from the First Nations and Inuit Health Branch (FNIHB), as well as from supplementary Parliamentary funding which may be provided throughout the fiscal year. The NIHB program registered 926,044 First Nations and Inuit clients in 2013 with a total budget in 2012/13 of $1.104 billion. Of total NIHB registered clients, 42,911 (4.6 %) were Inuit and pharmacy costs represented the largest proportion (41.9 %) of total expenditures at $462.7 million [11]. National level challenges to control pharmaceutical expenses include issues such as an aging population and increased reliance on pharmaceuticals [11]. Research indicates that due to higher levels of chronic disease, older individuals are prescribed more pharmaceuticals at higher costs than younger individuals [12]. Challenges specific to the NIHB program sustainability include population growth rates in its client base that are double that of the national average (Nunavut has the highest fertility rate in the country), and providing service to clients in remote and small communities [11]. The remoteness of Arctic communities provides unique challenges for pharmacy expenditure management (e.g. control of inventory levels) and oversight.

The NIHB Annual Report for 2012/13 indicates that pharmacy utilization rates - expressed as clients receiving at least one pharmacy benefit throughout the year as a proportion of the total number of eligible clients - are substantially lower in Nunavut (~45 %) than national NIHB averages (~62 %). In fact, Nunavut has the lowest utilization rates of all NIHB regions. This relatively low utilization rate, combined with a younger population than in other regions, is also reflected in the lowest per capita expenditures of all NIHB regions: $336 per capita in Nunavut compared to the NIHB average of $483 per capita. Average 2012/13 pharmacy expenditures in Nunavut were $718 per client, slightly lower than the NIHB national average of $750 per client. Total NIHB pharmacy expenditures in Nunavut were approximately $10.7 million in 2012/13. These costs increased 157 % between 2003 and 2013 (Table 1) [11].

**Table 1:** Federal non-insured health benefits (NIHB) and territorial Government of Nunavut (GN) pharmaceutical expenditures (in millions of Canadian dollars), in Nunavut, 2003–2013

NIHB annual pharmacy expenditures in Nunavut									
2003/04	2004/05	2005/06	2006/07	2007/08	2008/09	2009/10	2010/11	2011/12	2012/13
4.150	4.734	4.902	5.526	6.579	7.084	8.237	10.399	10.894	10.690
GN annual pharmacy expenditures in Nunavut									
2003/04	2004/05	2005/06	2006/07	2007/08	2008/09	2009/10	2010/11	2011/12	2012/13
Data unavailable							2.6	2.4	2.8

Territorial funding for the GN Pharmacy Program is through territorial budget allocation and supplemental funding as required. The Territorial Pharmacy Program focuses primarily on the acquisition and distribution of pharmaceuticals to all territorial health facilities; however, it also funds vaccines, blood services, contracted services and travel and transportation costs for leave replacement positions [13]. Vaccines in the territory comprise 48 % of total drug costs due to the high population growth rates (most vaccines are administered to children), and the availability of Synagis® (Palivizumab), a vaccine given to high risk infants to prevent Respiratory Syncytial Virus (RSV) infections. Synagis® comprises 45 % of total vaccine expenditures or 21.6 % of total pharmaceutical expenditures [13]. Total GN pharmacy expenditures in 2012/13 were approximately $2.8 million. These costs increased by 7.7 % between 2010 and 2013 (Table 1). Similarly, NIHB costs increased by 2.8 % during the same time period.

The overlay of social and economic marginalization with no-charge pharmaceuticals creates a landscape of both advantages and challenges requiring creative and adaptive policies and procedures for pharmaceutical service delivery. Ultimately, this landscape provides an important opportunity to provide pharmaceuticals where they otherwise might not be accessible, and to supplement health care services to improve health outcomes for the people of Nunavut.

### Pharmaceutical policy development

The mandate of policy is to regulate and govern the set of principles that guide decision making however, responsive health policy development is best facilitated through the routine input from front line service users and stakeholders [14]. Crucial information on the successes and shortcomings of policy application, or *how it works in real life*, must be available for adaptive and responsive policy development. There is an absence of published academic or governmental research regarding pharmaceutical practice in Nunavut despite considerable federal and territorial expenditures on pharmaceuticals and their importance with respect to health care delivery. Pharmaceutical health care for the people of Nunavut (referred to as Nunavummiut) is influenced by distinct Canadian federal and Nunavut territorial payment responsibilities for pharmaceuticals, as well as challenges in service provision to remote Arctic communities that are often affected by staffing shortages and isolation due to weather-related access issues. These factors introduce tensions into the acquisition, management and distribution processes which ultimately impact health care decisions. This research combines document analysis and interviews with patients, health care providers and policy makers in several communities in Nunavut to consider the research question of how existing pharmaceutical administration and distribution policies and practices in Nunavut impact patient care.

## Methods

This grounded theory community-based research and fieldwork was conducted between 2012 and 2014. The GN and community of Arviat invited research on pharmaceuticals due to concerns within the territory and specifically within the community as to policies and practices affecting patient care. Research planning and fieldwork was guided through consultation and assistance with the Qaujigiartiit Health Research Centre in Arviat and research associates who were long-term residents of Arviat. The topics and questions on the interview guide originated from community consultation during fieldwork in 2013 which were then edited and approved by the community prior to submission and further review by the Nunavut Research Institute for licensing approval. The diverse interests and concerns evident within the community and territory suggested that exploration of several issues was required. Therefore, this research follows a grounded theory framework and was designed so as to allow for the development of themes to emerge from the research itself [15].

Research methods include document analysis and semi-structured interviews. Document analysis includes review of federal and territorial policy documents and financial reports. Interviews were conducted in May 2014 with 35 participants in total, including residents/patients and health care providers from Arviat, health care providers and administrators from the Regional Health Centre and Qikiqtani General Hospital in Rankin Inlet and Iqaluit, and key policy makers in Iqaluit and Ottawa. Participants included CHC administrators, physicians, front counter staff, translators, CHNs working in various health care facilities, pharmacists and pharmacy technicians, community health representatives, and members of the Nunavut Pharmacy and Therapeutics Committee. Some participants provided interview responses based on multiple roles (e.g. as both health care providers and community residents). Participants were recruited for interviews through several methods including convenience sampling through information posted in several public locations in Arviat, local radio promotion inviting participation, snowball sampling, and purposive sampling for key informants. Interviews were conducted in private rooms, lasted a minimum of one hour in length and were audio recorded and transcribed for analysis. Research protocols received institutional approval through the University of Toronto’s Office of Research Ethics (Protocol # 28248) and were licensed through the Nunavut Research Institute (License # 01 033 13 N-M).

This research was designed to explore how existing pharmaceutical administration and distribution policies and practices impact patient care. In consideration of these research goals, interviews included open-ended questions regarding participants’ personal and professional (if applicable) experiences with pharmacy services (e.g. “Tell me about your experiences with medications”). Interview guides were developed with community, territorial and institutional assistance and questions were tailored for specific interview participant roles. For example, health providers were asked about the administration of their daily work while community members were asked about their personal experiences with medication acquisition and availability. Special care was taken to ensure language was appropriate to participant roles and simplified to reflect common language terms. Specifically, patient informed consent forms and questions were developed with institutional guidance from departmental researchers in populations with similar considerations of low educational and literacy levels; in Nunavut this can occur in both English and Inuktitut. Among Aboriginal adults in Nunavut, only 55 % have completed education above an elementary level [3]. Interview materials were translated into Inuktitut and participants were offered either English or Inuktitut documentation. Interpreters were made available on request for interviews. None of the participants elected for the use of an interpreter, and few selected the Inuktitut documentation. Verification and clarification was sought through iterative questioning until internally consistent understandings were observed. For example, distribution was explored throughout the supply chain and patient-provider encounters were verified from multiple informants on both sides of those transactions whenever possible.

QSR-NVIVO v10.2 [16] software was used to code and analyze data. The coding strategy developed for data analysis included using nodes to identify themes within the data and attributes to identify demographic information (i.e. ethnicity, role and community affiliation) from participants. The conceptual framework was informed by open coding, with emergent tree nodes outlining broad themes and child nodes allowing for more in-depth interrogation of the data. Queries on key words and themes were used to analyze the data and saturation was established when supporting evidence for findings was collected and found to be consistent from all participants within similar roles or with similar attributes.

## Results

Several key issues are consistently discussed among research participants and supported by document analysis. These issues involve: 1) the distinct NIHB and GN financial responsibilities for pharmaceuticals and their impact on health provider decisions for medication sourcing; 2) the resource requirements from CHCs to distribute retail pharmacy prescriptions through CHCs; and, 3) the financial losses and challenges associated with the return and disposal of unclaimed and expired medications.

### NIHB and GN financial responsibilities and medication sourcing

In theory, Inuk residents of Nunavut should be beneficiaries of some of the most complete medical coverage in Canada. While 10 % of Canadians report cost-related non-adherence to prescription medications [17], and 14 to 39 % (depending on province) of Canadians are uninsured for prescription medications [18], all beneficiaries of the Non-Insured Health Benefits program receive coverage for most prescription medications and many OTC medications such as antihistamines and acetaminophen [9]. In practice however, both the NIHB and the GN have been the subject of serious criticism stemming from federal audits of their administration and accountability for their respective programming, including pharmaceutical health care. The NIHB program has been criticized for serious oversights in monitoring of drug utilization and poor management and control of program expenditures, in particular the management of pharmacy benefits [19]. The GN has similarly been notified of serious concerns in regards to its financial management practices, lack of budget planning and consistent overspending, although notably, these issues were primarily attributed to the significant number of staffing vacancies and recognized difficulties in recruiting and retaining employees in the territory [20]. An example of the repercussions of the lack of GN financial management was the termination of a business relationship between the GN and a specialized pharmaceutical supplier due to vendor dissatisfaction. Both the NIHB and GN have recognized fiscal management issues that impact policy control and accountability, however lack of oversight may also hinder the examination of procedural discrepancies that are occurring in health care settings. These management issues affect administration and delivery of services throughout the territory and have the potential to impact patient care.

### Policy

Prescribing policies dictate that pharmaceuticals that do not meet the criteria for inpatient medications (including OTC) are to be prescribed and acquired through retail pharmacies. Remote communities often do not have full time health care providers with prescribing privileges such as nurse practitioners (NPs) and physicians in the community. Compared with Canadians overall, Aboriginal inhabitants in the territories are significantly less likely to have had contact with a General Practitioner or any other medical doctor in the past 12 months (58.8 % versus 78.7 %), and are significantly more likely to have had contact with a nurse than the average Canadian (49.0 % versus 9.8 %, respectively) [21]. Given that the majority of care in remote communities is delivered by nurses, Nunavut’s extended scope of practice allows CHNs and midwives to dispense many medications from wardstock [22]. In situations when a class of medication is unable to be dispensed by a CHN, prescriptions can only be obtained by communicating with the remote doctor on call.

### Practice

Divided federal and territorial financial responsibilities for pharmaceuticals create considerable tensions within the system between the NIHB and the GN. Health provider research participants discussed many factors influencing their decisions regarding medication sourcing (NIHB prescription or GN wardstock) including: pressures to shift expenses to maximize NIHB benefits, availability of a retail pharmacy in the community, weather, access to a prescribing health provider and staffing shortages.

Within CHCs, health providers are actively encouraged to minimize use of wardstock and maximize the use of NIHB benefits by writing prescriptions from retail pharmacies. One informant, a health provider in a community with a retail pharmacy, discussed a patient who had attended the CHC during regular business hours and was in need of a prescribed medication that was available in the CHC dispensary.

*“We have bottles of wardstock and the CHNs are allowed to dispense…[but] we would give them a prescription to go to the pharmacy…[administration] does not want us to [give the patient even a starter dose] because of budget…five years ago, they’d have gotten their meds from here”.*

In remote communities without retail pharmacies, weather delays and retail pharmacy dispensing times can delay pharmacy deliveries by anywhere from two to ten days. These delays in many cases would significantly impact patient care, and for this reason more medications are dispensed directly from wardstock and fewer prescriptions are written from retail pharmacies that would be expensed to the NIHB.

When a health care provider with prescribing privileges is unavailable within the community, CHNs are more likely to dispense a full course of medicine than to attempt to contact a prescriber outside the community for authorization, or to wait until a prescriber is available in person. Even in situations when a prescriber is within the community, it has been noted that understaffing (a common issue in the north) and/or high patient loads can cause CHNs to dispense medications from wardstock rather than seek out providers for prescription authorizations. A health care provider interviewed, referring to the GN’s Formulary Drug Treatment Codes categorizing medications which can be only initiated by a physician as “code B” medications, indicated that these are referred to by staff as “B is for Bother the Doctor”, while “code D” medications for which one dose may be dispensed by a CHN are referred to as “D is for Do It Yourself”. Health providers in remote locations are necessarily called upon to practice more independently and medication sourcing is but one of these areas of practice.

### Distribution of retail pharmacy prescriptions through community health centres

In the majority of Nunavut communities, primary care is delivery by nurses with physicians only visiting sporadically based on rotating schedules and weather-related accessibility. Staff turnover for remote nursing is recognized as a serious challenge, with vacancy rates between 37 and 57 % across the regions in Nunavut [23]. These nursing shortages have been noted to result in an increased reliance on casual nursing staff with decreased familiarity with Inuit culture, a shift from primary health care to more emergent acute and chronic care, and increased stress levels on nurses leading to decreased job satisfaction, and burnout [23, 24]. Nurses practicing under an extended scope of practice take on duties that southern nurses in more urban centres are not asked to perform, with routine staffing shortages further exacerbating the situation. Health care informants indicate that they are often called upon to take on increasing levels of administrative work, such as the distribution of retail pharmacy prescriptions, which take time away from patient care.

### Policy

In communities without retail pharmacies, many of the administrative roles of pharmacists (i.e. stocking, distribution of medications to patients and patient counselling), are performed by CHCs. When medications from retail pharmacies arrive at the CHC, staff are required to verify shipping records and then provide a list to administrative staff for patient notification. Front counter staff then make attempts to contact the patient (generally by phone) to inform them that their medication has arrived. Unpacked medications are to be shelved in an organized manner (alphabetically by packaging type) in the CHC dispensary to await patient pickup.

Many chronic medications are prescribed with automatic refills, indicating that retail pharmacies send out one month supplies of medication at regular intervals without further instruction required until the prescription expires. There is currently no system in place to inform the retail pharmacy if the current prescription has been picked up, therefore pharmacists proceed with the assumption that the next month’s supply of medication is required, even if a previously unclaimed prescription is still shelved in the CHC dispensary.

### Practice

When understaffing and high patient loads occur, duties associated with retail pharmacy deliveries may be deprioritized. Many health care provider informants voice displeasure and frustration with the additional administrative burdens of these deliveries, which they see as taking time away from patient care. As one health provider informant stated,

*“there’s boxes and boxes and boxes, I’m talking hundreds of prescriptions a week, that are filtering through this Health Centre …if I had to sit every day and figure out people’s phone numbers, most people have no phones, some people are nomadic, they’re moving from house to house, it’s very difficult to track them down to say ‘hey, your medications are here’.”*

Community participants speak of not receiving notification that their prescriptions have arrived and visiting the CHC several times to make enquiries, sometimes to discover that their medications had arrived a week or more earlier. As two patient informants voiced,

*“we’ve had prescriptions sit at the Health Centre, for a week or two, longer than they should, and we were waiting…so they’re in the community, and they haven’t called us. They’ll say ‘this came in last week; we didn’t know you were waiting for it’.”*

Many health care provider participants note that there are a large number of patients who never retrieve these medications. While lack of awareness among patients that their medications have arrived is one possible explanation, health provider informants suggested several other possibilities including: lack of understanding for the rationale for the medication, denial of their medical diagnosis or the severity of potential outcomes of non-adherence and distrust of medications. As the vast majority of Nunavummiut speak Inuktitut as their first language and few health care providers are fluent, language challenges may also contribute to misunderstandings [25]. Family pressure can also impact adherence, for example a health care provider informant described an incident when they asked a patient diagnosed with severe depression if they were taking their medications, and they responded,

*“my grandmother said that I am not mentally ill and ‘you’re not taking that medicine’.”*

Informants suggest another possible factor might be the lack of understanding among many Inuit as to the high cost of medications. Inuit are life-long recipients of pharmaceuticals provided with no patient co-pay, including such OTC medications as acetaminophen and ibuprofen. Even for most medications received from retail pharmacies, there is no cash value printed on the receipt. Informants suggested that an understanding of the financial value of the medications that are being prescribed might influence adherence by Inuit patients. As one health provider informant, with over 7 years of service in Nunavut discussed,

*“with the HPV vaccine, we were having really low impact and one of the conversations that I started to have was ‘do you realize that people have to pay for it and that and [we] are now offering it for free?’ , and then people were kind of like ‘oh’, like it kinda got their attention. Not that it’s going to keep your daughter from having cancer…but when it’s free! [referring to medication]…maybe if they don’t see the value in it, they don’t bother or they throw it away or something like that”.*

Through what is likely a combination of these factors, virtually all participants consistently note that many retail pharmacy medications are not getting to, or being used by, the intended patient.

**Fig. 1:**
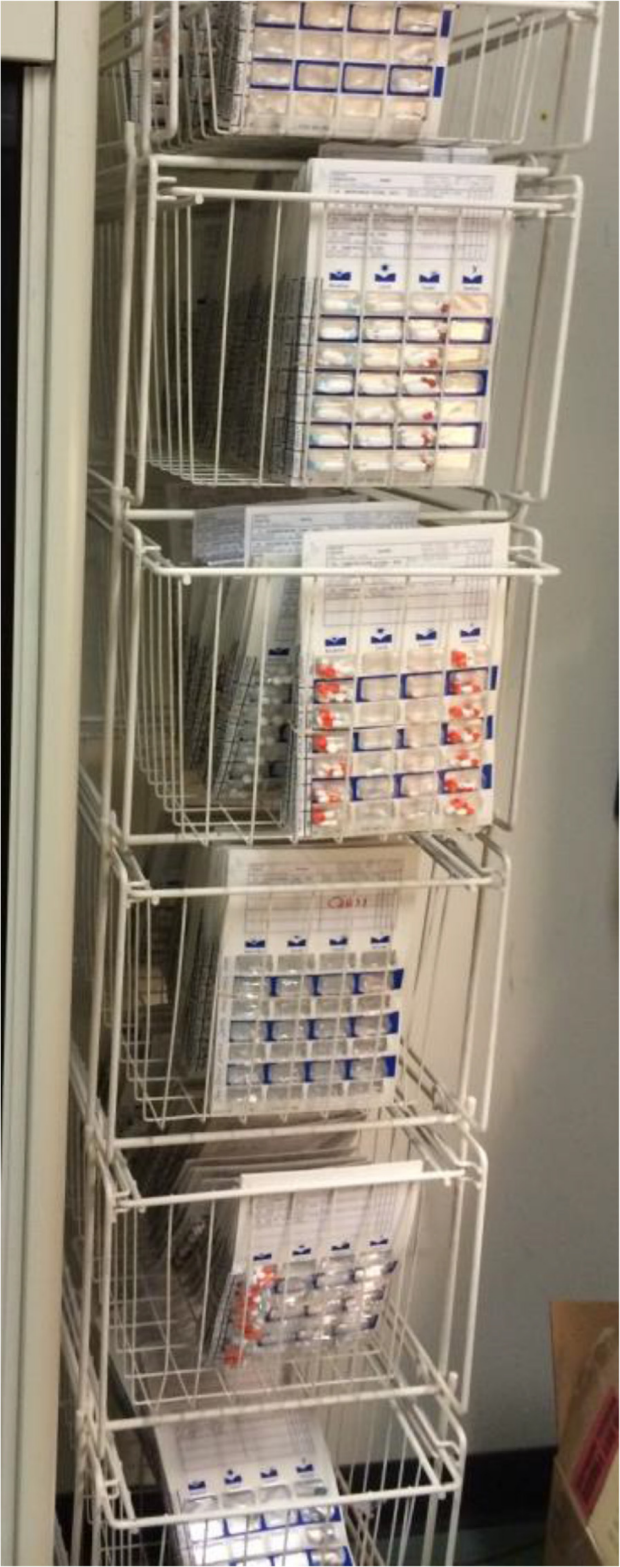
Racks of convenience packaging (i.e. blister packs) of medications awaiting patient pickup in the Community Health Centre (CHC) dispensary. Storage and organization of these large volumes of medication is a challenge. (Photograph by Sandra Romain)

**Fig. 2:**
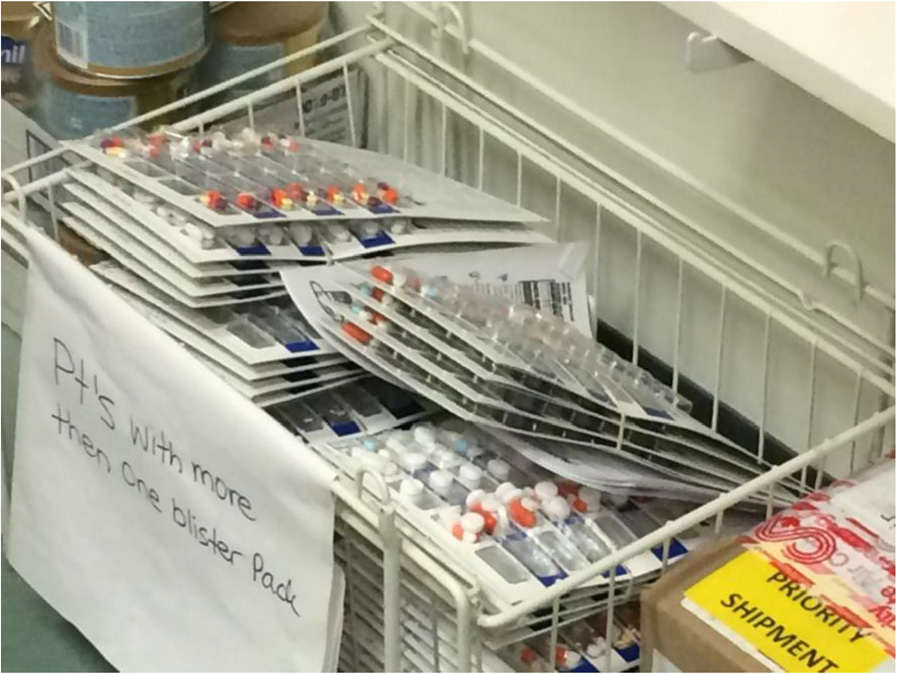
Bundles of medications in convenience packaging (i.e. blister packs) awaiting patient pick-up in the Community Health Centre (CHC) dispensary. Each convenience pack contains medications for one week. Many medications arrive in communities dispensed with sufficient medication for one month. This is shown in the bundles of four blister packs. (Photograph by Sandra Romain)

**Fig. 3:**
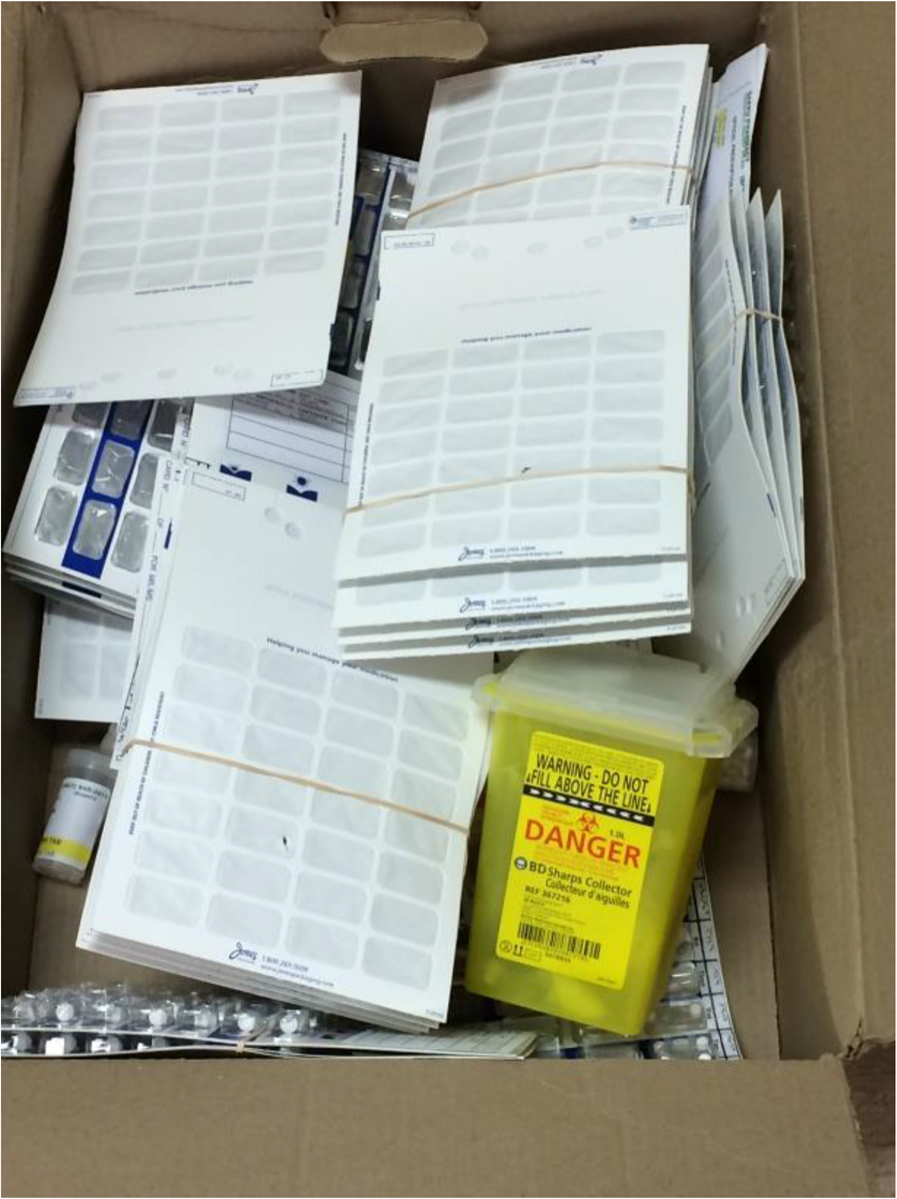
A box of unclaimed retail pharmacy medications, the majority in convenience packaging (i.e. blister packs), awaiting return to the Regional Health Centre for disposal through incineration. Some informants indicated that medications might be returned in medical sharps containers (seen bottom right) to facilitate disposal. (Photograph by Sandra Romain)

Unclaimed medications were observed in the CHC dispensary in overflowing boxes and shelving units (Figs. 1, 2 and 3). Several patients were observed to have multiple prescriptions dating back three of four months. Many health providers voice frustration with this issue and the economic wastage incurred. As one health care provider informant explained,

*“The problem is, when a physician writes a prescription, commonly three, four, six refills may go on that prescription…sometimes we get a backlog…the other day I noticed that we had a four month supply of a medication for a child who I knew was no longer even in the community. This is a problem, it is a wastage of medication, medications are having to be destroyed, at least once a month there is a box full of medications, for various reasons…hundreds of thousands of dollars worth in loss…I calculated there not too long ago that one certain load of medication came in from a household and there was nothing short of about 12 to 15 thousand dollars worth of medication. This is a problem.”*

Although there is some confusion as to where this notification chain breaks down, the effects of this disorganization can negatively impact patient care through loss of trust in the CHC, as two community informants indicated:

- *I1: in the bubble packs…a lot of people who have chronic illness have bubble packs, right? They are trying a new medication, or they’re bumping up a milligram, they’ll be on it for a long time and not understand why when ‘the doctor said I should be off it by now and I’m still on it’*
- *SR: So the doctor is saying “we’re going to discontinue on this”?*
- *I1: oh, yeah*
- *SR: but the next blister pack that arrives…*
- *I1: yes*
- *SR:still has the pills in it?*
- *I1: yep*
- *SR: Now what does the person do? Just takes it?*
- *I1: usually*
- *SR: just assumes, “oh, I guess I misunderstood?”*
- *I1: yep*
- *I2: or they start to mistrust the Health Centre and they stop taking all their pills all together*
- *I1: oh, yeah - that’s a big one*

Many health providers voice the most frustration with the lack of an effective mechanism to stop retail pharmacies from filling repeat prescriptions when the patient has not yet picked up the previous prescription. This is stated to be the greatest source of multiple unclaimed prescriptions for the same individual being stored in the dispensary. Several practices have been used to minimize repeat unclaimed prescriptions, although none are implemented broadly across the territory or with proven results. One health provider informant indicates that they now issue repeat prescriptions with a written notation of “as requested by patient”, to necessitate a patient initiated refill. One CHC in the Kivalliq provides a phone at the front desk to actively encourage patient calls to the pharmacy at the time of pick-up so that the patient can request their next month’s prescription be filled. A key pharmacy informant mentions a corresponding increase in patient calls from this community without solicitation, although the informant is unaware as to the reason why this community had noticeably more calls than others. One health provider informant is familiar with a double label system used in other jurisdictions (e.g. Labrador), whereby the retail pharmacy prints two labels for each retail pharmacy prescription and when patients pick-up their medications, one label is removed, affixed to a reorder sheet and faxed back to the pharmacist to inform them that the patient has received the medication and they are authorized to refill the following month’s prescription. While some health provider informants are enthusiastic about these various interventions, others voice concerns about the labour intensive administrative process and increasing responsibility for medication management on the part of CHC staff.

The substantial and sustained requirements of the already taxed CHCs and CHNs to organize, store and distribute retail pharmacy medications likely contributes to the large quantity of unclaimed medications which are indicative of patient non-adherence and suboptimal pharmacotherapy.

### Return and disposal of unclaimed and expired medications

Improper pharmaceutical waste management can have significant health, criminal and environmental impacts. In relation to health and safety, most medications are still considered safe beyond their expiration dates, however some (e.g. nitroglycerin, insulin, liquid antibiotics, epinephrine pens) lose efficacy and given the emergent need for these drugs during life threatening situations, potency is mandatory [26]. Criminally, recreational misuse of illegally obtained pharmaceuticals, often from family medicine cabinets or friends, is a growing trend in Canada resulting in increases in theft and fraud as well as health impacts such as overdose deaths, suicides and emergency care costs [27, 28]. Environmentally, pharmacologically active substances have been detected in surface and ground water at levels rivalling some pesticides [29]. This is especially problematic given the environmental sensitivity of arctic ecosystems and the heavy reliance on subsistence hunting of both land and sea mammals.

### Policy

Some medications purchased for wardstock by the GN may expire prior to dispensing in the CHCs. As part of regularly scheduled inventory within CHC dispensaries, wardstock is to be shelved in consideration of expiration dates (i.e. newer stock at the back) and any expired medications are to be removed to ensure patient safety. Some expired medications may also be eligible for reimbursements from manufacturers or distributors. According to a key pharmacy informant, some injectable or intravenous drugs may be eligible for reimbursements of as much as $2,700 for a single expired dose.

Expired wardstock medications that are ineligible for reimbursement and unclaimed retail pharmacy medications require environmentally safe disposal and may also be subject to control protocols (e.g. narcotics). The proper disposal of pharmaceuticals in Nunavut is governed by the GN Narcotic and Controlled Drugs Policy (revised edition released Fall 2014) which regulates the “acquisition, storage, prescribing, administration, record keeping and disposal of narcotic and controlled drugs” as well as the responsibilities of authorized health care providers who work with these substances, and by the federal Controlled Drugs and Substances Act [30]. Controlled substances entering the community are closely monitored with pill counts, sealed shipping envelopes and double signatures. However, if these medications are unclaimed or expired, only GN wardstock narcotic and controlled substances are subject to the same procedures in reverse when leaving the community; retail pharmacy medications are subject only to routine disposal. Unclaimed retail pharmacy medications and expired wardstock are sent to Regional Health Centres for incineration or (less frequently) to retail pharmacies for waste management.

Unclaimed retail pharmacy medications that are not picked up may also be eligible for reversal of NIHB expenses through a procedure to reabsorb costs while continuing to remunerate pharmacists for their dispensing fees. Retail pharmacies have varied requirements for accepting these returns. One key pharmacy informant indicates that these reversals are only possible for unclaimed medications that would have been dispensed directly from the retail pharmacies (i.e. to in-town clients) as they are able to confirm the environmental conditions of medication storage, as well as ensure that the medication has been kept in a secure location and has not been subject to tampering. Unclaimed medication reversals are not accepted by some retail pharmacies when medications are dispensed for use in remote communities. Yet another key pharmacy informant indicates that the supply chain from pharmacy to CHC and back was sufficiently controlled to allow for a reversal, as it was secure enough for narcotic and controlled substances protocols. When unclaimed retail pharmacy medications are unable to be returned to retail pharmacies, CHCs are instructed to send them to Regional Health Centres with expired GN wardstock for incineration.

### Practice

In speaking to many community member participants about what they do with their personal unused medications, most indicate that they throw them in the garbage. In recognition of this issue and as confirmation of its significance, one health worker informant describes a door-to-door “spring clean up” program that was organized for collecting household medications; this initiative collects several large garbage bags annually requiring the use of a pickup truck due to weight. These findings indicate that there are likely large quantities of unused medications in community homes that would benefit from assisted disposal programs.

Within the dispensary at the CHC, large boxes of unclaimed medications require frequent disposal services. Participants provide varied and ambiguous responses to enquiries regarding this process, alternatively indicating that medications are shipped in sealed cardboard boxes or that they are deposited individually into medical sharps containers and sealed before shipment (Fig. 3). This latter process requires the time consuming process of removing individual medications from the many unclaimed blister packs. Many interview participants describe the time consuming and necessarily deprioritized efforts to routinely return the large quantities of medications for destruction. Time lapses between shipments are estimated to be between one and three months in duration, depending on staffing and storage capacity.

Several interview participants at both the provider and administrative levels discuss the practice of the transfer of retail pharmacy medications into wardstock inventory. Participants explain this practice as being done through either front-end or back-end restocking. If a CHC dispensary does not have stock of a medication that is needed immediately and one is available in the storage units holding retail pharmacy medications that have not yet been picked up, they might dispense the retail pharmacy medication to the patient in immediate need. One patient informant described their experience,

*“we’ve gotten something out of the dispensary and it will have somebody else’s label already on it from [retail pharmacy name]…so they’ve taken a labeled drug from [retail pharmacy name] and put them in the dispensary and relabeled it to give it to us.”*

This front-end substitution may leave the patient for whom the medication was specifically intended without the medication, but informants note that either the medication is replaced with GN wardstock when it arrives or that more frequently, the medication is taken from the extensive supply of unclaimed retail pharmacy medications that would eventually need to be incinerated. This practice is stated to be extensive enough as to reduce budgetary requirements for CHC inventory. As one health administrator informant states,

*“If I had twenty patients that I was getting prescriptions in monthly that they never pick up, and I put them in my stock, and use them to dispense, I’m at zero budget…I’ve been in communities where they are doing it”.*

An alternative form of this medication substitution is the replacement of medications already dispensed from wardstock with retail pharmacy medications. This back-end substitution is used when a medication is dispensed from wardstock that might otherwise be prescribed from a retail pharmacist, but that due to timely need (e.g. impending weather) is dispensed directly from the CHC. The prescription for the patient is then sent to the retail pharmacy and when the medication arrives, it is used to replace the GN wardstock that has already been previously dispensed. Although these practices have significant budgetary and possibly legal implications, the health provider informants interviewed are most concerned with patient-centred care and are focusing on the urgency to fill an immediate patient need for medications that could take days or weeks to arrive.

Practices at this time do not track or inventory the destruction medications, including retail pharmacy controlled substances such as narcotics. This lack of oversight could foreseeably result in the unlawful removal of controlled substances by unauthorized individuals. Health provider informants at several levels are concerned that although unclaimed retail pharmacy narcotics are kept in a secure, separate location from other non-controlled medications in the CHC dispensary, that there are few procedures in place to ensure the unclaimed narcotics are destroyed lawfully and appropriately. While the protocol for the destruction of expired GN wardstock controlled substances is compliant with the Controlled Drugs and Substances Act, several key informants indicate that there is considerable concern and debate among stakeholders as to the legal and financial responsibility for the disposal of retail pharmacy narcotic and controlled substances. At the centre of the controversy is confusion about ownership and authority over a prescribed medication intended for an individual who has never taken possession. However, the lack of current oversight in regards to their destruction presents a significant risk for the “disappearance” (i.e. unlawful removal) of these medications. These issues are currently under consideration by the GN Pharmacy and Therapeutics Committee in consultation with the NIHB and legal counsel to ensure the development of a policy to address this issue.

## Discussion

A number of key themes emerge from the interviews conducted for this research: 1) the tensions between NIHB and GN financial responsibilities influence the decisions of health providers and may affect patient care, 2) significant human resources are utilized in Community Health Centres to perform distribution duties associated with retail pharmacy medications; 3) large quantities of unclaimed prescription medications are suggestive of significant financial losses, suboptimal patient care and lower adherence rates; and, 4) the absence of a clear policy and oversight of some controlled substances, such as narcotics, leaves communities at risk for potential illegal procurement or abuse.

### Theme one: NIHB versus GN financial responsibilities for pharmaceuticals

As the NIHB is a federally administered program responsible for multiple jurisdictions, Nunavut may be subject to less direct review of expenditures compared to other jurisdictions due to its low pharmacy utilization rates, lowest per capita expenditures and lowest overall pharmaceutical costs. These low figures, combined with past claims of program mismanagement and lack of oversight may be contributing factors in some of the practices identified by research participants leading to unnecessary repeat prescriptions and unclaimed medications. Concurrently, due to the GN’s recognized staffing issues and financial mismanagement in the Department of Health and Social Services, concern regarding the oversight of pharmaceutical expenses may be reasonable.

As CHC administrators are evaluated on their ability to balance their budgets inclusive of wardstock pharmaceutical costs, this may contribute to the practice of inventory transfer from NIHB medications to GN wardstock when retail pharmacy medications are available and unclaimed. Additionally, frustration with the losses associated with quality medications going unclaimed and heading for incineration, and/or with insufficient inventory levels to reflect isolated communities may influence health provider decisions to use retail pharmacy medications for CHC patient care. Systemic policy modifications might reduce the necessity to transfer stock from the NIHB to the GN through increased efficiency in inventory management systems.

Decision making for front line health providers is complex and often involves many considerations that are beyond merely following policy guidelines. Ultimately what is most prominent in interviews is decision making that is patient-centred above other considerations. However, increased situational pressures (such as isolation and staffing shortages) on CHNs to independently dispense medications from GN wardstock may reduce pharmacy industry recommended independent double-checks and increase the potential for dispensing errors. Policy development that recognizes the complexity of the medication sourcing decision-making process may support health providers in their focus on patient care.

### Theme two: Pharmacy duties of Community Health Centres

Research participants working in CHCs repeatedly voice frustration and displeasure for the duties and responsibilities associated with the distribution of retail pharmacy medications. Many felt that the role and associated tasks of a pharmacist were being forced on the CHC when understaffing was a common concern. Their inability to perform the time consuming task of notifying patients that their medications were available for pickup is a source of irritation and frustration. Informants recognize that patient non-adherence is likely impacted by an inability for patients to pick up their medications, but also impeded by staffing capacity to notify patients and organize and distribute medications. None of the participants suggest that they are “above” the task, but identify it as an impediment to seeing patients and their many other duties.

Many patient participants also voice frustration with the deprioritized notification of medication arrivals or loss of their medications due to the challenges of storing and organizing large volumes of patient-specific medications in the CHC dispensary. Several interview participants in Arviat strongly suggest that a retail pharmacy is needed in the community and that the population size (~3000) supports the investment, identifying that it would release the CHC from its current responsibilities for the distribution of prescriptions. Overwhelmingly, all interview participants – health providers and patients - are dissatisfied with the current distribution system within CHCs for prescriptions arriving from retail pharmacies outside of the community. This issue affects the majority of communities in Nunavut, as only three of the twenty-five communities currently have retail pharmacies. Not all communities have population sizes that would support a retail pharmacy, however many health care informants express interest in exploring other options, such as the inclusion of a pharmacy technician on staff at CHCs to take on a distinctly tailored role including specific administration, inventory and distribution duties.

### Theme three: Losses associated with unclaimed prescriptions

The administrative challenges of CHC distribution practices are likely reflected in the large number of unclaimed prescriptions. Unclaimed medications result in significant loss of human resource capacity through repeat attempts to contact patients, time taken to package up and return medications for disposal, return shipping costs and incineration. These unclaimed medications also signify substantial financial losses as NIHB funded medications are not being used for patient care.

Retail pharmacy policy that is unreceptive to the return and NIHB expense reversal of unclaimed medications may reflect safety concerns, but there is also minimal financial motivation to reabsorb stock or to rapidly respond to prescription discontinuation requests. While pharmacies that accept the return of unclaimed medications are still entitled to claim a dispensing fee from the NIHB through a special code for this purpose, they are effectively performing two distinct tasks (i.e. dispensing and then reabsorbing medications back into stock) and being reimbursed for only one. Additionally, the pharmacy loses the original sale and may have to consider expiration dates and/or if the returned product is resalable.

Several strategies to minimize automatic prescription repeats are suggested but untested. Further examination into the efficacy of these different approaches might provide sufficient evidence to standardize a method to reduce these losses across the territory. If similar issues of repeat and unclaimed prescriptions are found in other jurisdictions nationally, the savings to the NIHB could be quite substantial.

What is repeated vehemently among many health provider informants is their disbelief that this long term wastage is apparently going unnoticed and that auditing or regulatory bodies have not identified these losses and found a way to minimize their occurrence. This lack of oversight might be better understood in light of the Auditor General’s Report on the NIHB, and Nunavut’s relatively low per capita pharmacy utilization rates, however this area of concern would benefit from further examination. Policy development that recognizes the limited staffing in CHCs and the need for enhanced communication with retail pharmacies may facilitate medication delivery to patients and reduce the financial losses attributed to unclaimed medications.

### Theme four: Controlled drugs oversight and potential for illegal procurement

As an associated result of the excessive unclaimed prescriptions, the potential for the illegal procurement of narcotics and controlled substances is highlighted as a potential threat to the community. Health provider informants are genuinely concerned and looking for guidance to minimize this risk. The absence of policy and oversight on this issue presents an opportunity for narcotics to be abused or available for illegal trade. Interviews demonstrated that there is an urgent need for this risk to be mitigated through a clear policy that is compliant with the federal Controlled Drugs and Substances Act. Policy makers are struggling with these issues currently, to minimize the great risks of narcotics that can go unaccounted for with little to no notice. This timely issue poses great risks to communities every day that a clear policy is not available and consistently administered.

## Conclusions

This research identifies several areas of concern which may prove beneficial in providing direction for future policy development that best serves the needs of Nunavummiut: 1) NIHB and GN financial responsibilities for pharmaceuticals and their effects on medication sourcing, 2) resource requirements of CHCs to distribute retail pharmacy medications, 3) human resource, patient adherence and financial losses associated with unclaimed medications, and 4) community risks associated with the absence of clear policy for the disposal of controlled substances. The financial, health, safety and efficiency issues identified in this research require consideration through policy development that is familiar with the many challenges of service provision in Nunavut. Demographic factors such as a young and rapidly growing population combined with geographical, sociological and environmental challenges affect the selection and the availability of medications where and when they are required. The tensions created by the competing NIHB and GN financial responsibilities affect everyone from the GN policy writers to the Inuit child in need of an out-of-stock inhaler in a storm-isolated community.

Health providers in remote communities make decisions first and foremost based on patient care, but often these decisions put their actions in direct conflict with policy and procedures that may not be reflective of the realities of stock shortages and dispensing challenges in a small, remote community. Given that providers are most aware of both the needs of quality patient care and the policy restrictions that create challenges in meeting this demand, future policy development should be considered that is reflective of this knowledge and minimizes the need for providers to make decisions that may fall outside of accepted policies and procedures.

Through addressing these identified issues in future policy development, several benefits may be possible: financial savings may be realized through minimizing pharmaceutical wastage, community safety may be improved through the proper administration and disposal of medications including controlled substances, and adherence may increase through consistency, availability and accuracy of medications. Participants in this research, who live and work in Nunavut, have been best suited to identify issues in need of attention, and are also positioned to benefit the most from policy development which addresses their concerns.

## Abbreviations

CHC: Community Health Centre
CHN: Community Health Nurse
CWB: Community Well-Being Index
FNIHB: First Nations and Inuit Health Branch
GN: Government of Nunavut
NIHB: Non-Insured Health Benefits
NP: Nurse practitioner
OTC: Over the counter
